# Continuous Recirculation of Microdroplets in a Closed Loop Tailored for Screening of Bacteria Cultures

**DOI:** 10.3390/mi9090469

**Published:** 2018-09-17

**Authors:** Pawel R. Debski, Karolina Sklodowska, Jacek A. Michalski, Piotr M. Korczyk, Miroslaw Dolata, Slawomir Jakiela

**Affiliations:** 1 Department of Biophysics, Warsaw University of Life Sciences, 159 Nowoursynowska Street, 02776 Warsaw, Poland; pawel_debski@sggw.pl (P.R.D.); karolina_sklodowska@sggw.pl (K.S.); 2 Department of Plant Genetics, Breeding and Biotechnology, Warsaw University of Life Sciences, 159 Nowoursynowska Street, 02776 Warsaw, Poland; 3 Faculty of Civil Engineering, Mechanics and Petrochemistry, Warsaw University of Technology, 17 Lukasiewicza Street, 09400 Plock, Poland; jacek.michalski@pw.edu.pl; 4 Institute of Fundamental Technological Research, Polish Academy of Sciences, Pawinskiego 5B, 02106 Warsaw, Poland; piotr.korczyk@ippt.pan.pl; 5 Department of Econophysics and Physics Application, Warsaw University of Life Sciences, 159 Nowoursynowska Street, 02776 Warsaw, Poland; miroslaw_dolata@sggw.pl

**Keywords:** microfluidic loop, bacteria cultures, screening, antibiotic treatment, *Escherichia coli*

## Abstract

Emerging microfluidic technology has introduced new precision controls over reaction conditions. Owing to the small amount of reagents, microfluidics significantly lowers the cost of carrying a single reaction. Moreover, in two-phase systems, each part of a dispersed fluid can be treated as an independent chemical reactor with a volume from femtoliters to microliters, increasing the throughput. In this work, we propose a microfluidic device that provides continuous recirculation of droplets in a closed loop, maintaining low consumption of oil phase, no cross-contamination, stabilized temperature, a constant condition of gas exchange, dynamic feedback control on droplet volume, and a real-time optical characterization of bacterial growth in a droplet. The channels (tubing) and junction cubes are made of Teflon fluorinated ethylene propylene (FEP) to ensure non-wetting conditions and to prevent the formation of biofilm, which is particularly crucial for biological experiments. We show the design and operation of a novel microfluidic loop with the circular motion of microdroplet reactors monitored with optical sensors and precision temperature controls. We have employed the proposed system for long term monitoring of bacterial growth during the antibiotic chloramphenicol treatment. The proposed system can find applications in a broad field of biomedical diagnostics and therapy.

## 1. Introduction

Since its emergence in the early 1980s, microfluidics has expanded rapidly as a multidisciplinary area of scientific and technical research, covering the fluid dynamics at the microscale and its various applications in biology, chemistry, and medical diagnostics [1,2]. The small volumes of fluids contained in droplets are large enough to be fully functional chemical reactors [3,4], making the chemical processes unaffected by downscaling [5].

The robust development of biochemical and clinical applications [6,7,8] of microfluidic technology prompts the development of novel microdevices that make the microfluidic systems even more versatile. Current microfluidic devices should not only operate on small amounts of reagents, but also be portable, precise, cost-effective, programmable, and offer new capabilities to carry out a variety of laboratory operations, either subsequently or simultaneously (in parallel) [2,9]. This approach is widely used in modular microfluidic systems, such as swappable fluidic modules [10,11,12,13], a Lego-like modular microfluidic platforms [13,14,15], and 3-D modular microfluidic devices [16,17,18]. They are all designed for biological and chemical applications. 

Microfluidic techniques are a powerful tool and are currently used for cell studies. They enable high-throughput analysis and precise manipulation of reagents in the vicinity of cells [19]. Manipulation of droplets is possible due to high-quality droplet generations, their merging, analysis, mixing, and sorting [4,20,21,22]. Both mammalian cells as a single cell analysis [23] and bacteria as cell cultures [4] are currently studied in droplet microfluidics. Using microfluidic devices, simple monodisperse droplet generation with cells were developed [24,25] through single-cell analysis, as well as [19,26] selective encapsulation and [27,28,29] long-term incubation of cells [1,22,30].

Moreover, in the literature, we can find classical microfluidic loops (a microchannel splitting into two branches, which are combined next) designed to study the chaotic dynamics of flowing droplets [31,32], as well as a loop reactor assembled to conduct a multistep reaction with the aqueous phase [33].

We thus present a versatile capillary-based fluidic system, which allows for: (a) closed-loop droplet recirculation with low consumption of the continuous phase, (b) no cross-contamination and dynamic feedback control on droplet size, (c) integrated real-time optical characterization of droplet size and bacterial growth in a single droplet, (d) a minimization of the risk of liquid spillage to the environment. Unlike other solutions, the examined drops in the presented system move all the time during the incubation process. The continuous phase is not waste, but it is in continuous recirculation; this distinguishes the presented solution from others presented in the literature. The only oil consumption (1 mL for the entire screening) during the experiment occurs when pulling out the droplets for further analysis. Other solutions presented in the literature suggest a waste of the oil phase when correlated with flow rate, and are ordered in ~mL per hour [4,22,34]. Moreover, our system of continuous droplet recirculation sorts out the most challenging cross-contamination problem [35] between droplets and oil as well as droplets and the surface of the microfluidic device.

## 2. Materials and Methods

### 2.1. Reagents

The continuous phase consisted of hydrofluoroether 7500 (HFE-7500) (Semicon, Warsaw, Poland) with a 3% *w*/*w* of 1*H*, 1*H*, 2*H*, 2*H*-Perfluoroctanol (Sigma-Aldrich, Poznan, Poland), a viscosity of μ_o_ = 1.24 cP (1 cP = 1 mPa·s), and an oxygen solubility larger than 100 mL of gas per 1 L of liquid at 1 bar of air pressure at 35 °C [36]. 

The dispersed phase consisted of fresh Luria-Bertani Broth (LB Broth, Biocorp, Warsaw, Poland) and the suspended bacterial culture strain *Escherichia coli* (ATCC 35218). The prepared stock solution of microorganism contained 30% glycerol (Sigma-Aldrich, Poznan, Poland) and was stored at −80 °C. At the beginning of experiments, cells were streaked on LB agar plates, which were then incubated overnight at 37 °C. After that, an individual colony was used to inoculate fresh LB medium, which was placed for overnight incubation at 37 °C and shaking at 200 rpm. Finally, aliquots were transferred to fresh LB media and grown until the absorbance at 600 nm reached 0.1. The culture was diluted tenfold before transferring into droplets. 

The antibiotic stock of chloramphenicol (Roth, Warsaw, Poland) was prepared before experiments using 50% (*v*/*v*) aqueous solution of ethanol. The initial concentration of stock was 20 mg/mL. Next, the solution was sterilized with a syringe filter (Celltreat, Mixed Cellulose Ester (MCE) membrane, 0.22 μm pore size, 30 mm diameter). Before conducting the experiments, we diluted antibiotic stocks in LB broth to the concentration of 10 μg/mL and transferred the drug solution to the inlet of our device. The concentration of the antibiotic was subsequently dosed during the merging of droplets in Teflon fluorinated ethylene propylene (FEP) tubing.

A solution of 10% (*v*/*v*) red (Watchman, Warsaw, Poland) and water was used to observe the circulation (Video S1) of droplets in a loop.

### 2.2. Microchip Fabrication

**Figure 1:**
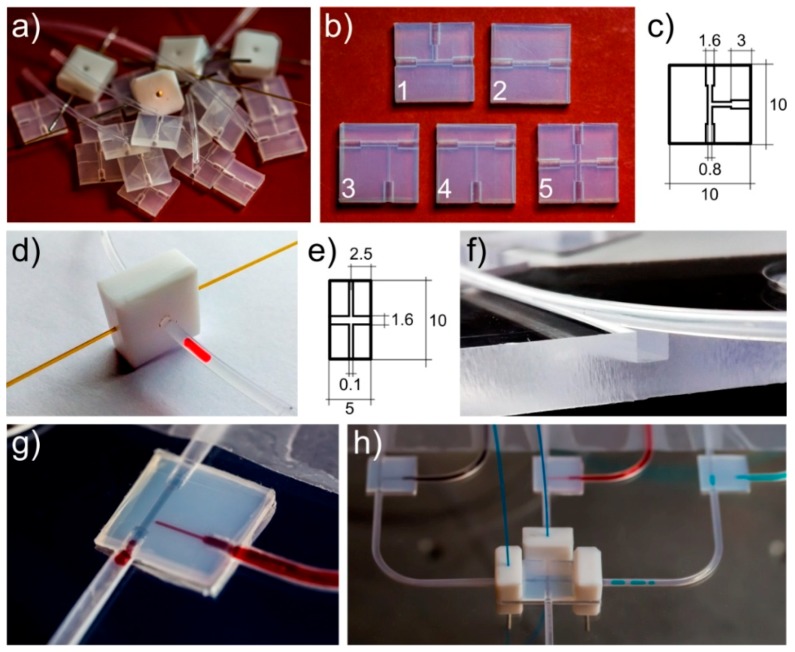
(**a**) Drilled/milled simple connectors, holding fibers and tubes. (**b**) Five different connectors: three T-junctions (1, 3, 4), a cross (X)-junction (5), and an adapter (2). (**c**) Dimensions of the T-junction unit. (**d**) The sensor cube that supports optical fibers connected with a light source and a photodetector designed for absorbance (optical density) measurements. (**e**) Dimensions of the sensor cube. (**f**) Polycarbonate frame (supporter) for the microfluidic system (support for tubing shown). (**g**) Assembled and operating T-junction—we used three tubes and one T-junction unit. (**h**) Droplet-on-demand section of the system that enables merging droplets from three T-junctions. Teflon holders (white drilled cuboids) support fibers to detect the position of a droplet to stop/switch the flow in the tubing at the appropriate time.

The proposed microfluidic chips were based on the capillary tubing (Teflon FEP tubing, Dolomite, Royston, UK, internal diameter 0.8 mm, outer diameter 1.6 mm) and three types of components (cubes): T-junctions, X-junctions, and adapters (Figure 1a). Cubes were 10 mm wide, 10 mm long, and 3 mm thick and were prepared in a Teflon FEP sheet [12] (Figure 1b). They were entirely matched by drilling with the applied capillary tubing (Figure 1c). In order to ensure mechanical stability of connections between the Teflon FEP capillary tubing and connectors, the diameters of milled holes in the connectors were 0.04 mm smaller than the outer diameter of the tubing. 

Moreover, to measure the absorbance—or the optical density—in droplets, as well as the lengths of droplets and the distance between them, we used custom-made cubes of Teflon with two perpendicular through-holes (Figure 1d,e). The diameter of the through-holes was fit to the outer diameter of the tubing. In turn, the second through-hole was fit to the optic fiber (FG105LCA, Thorlabs, Newton, NJ, USA). The optic fibers were used to connect with an external optical photodetector (TSL257 family, Taos, Plano, TX, USA) and the LED source of light (HMIB-44WY-TR7, Huey Jann Electronics, Taichung, Taiwan). That component can be freely placed along the tubing, enabling to mount it at different positions (Figure 1h).

To immobilize the system and fix the positions of optical sensors, we applied the polycarbonate frame (supporter) (Figure 1f). The supporter had a 1.6 mm wide square cross-section channel, which was milled for tubing and had appropriate holes cut for connectors and sensors.

**Figure 2:**
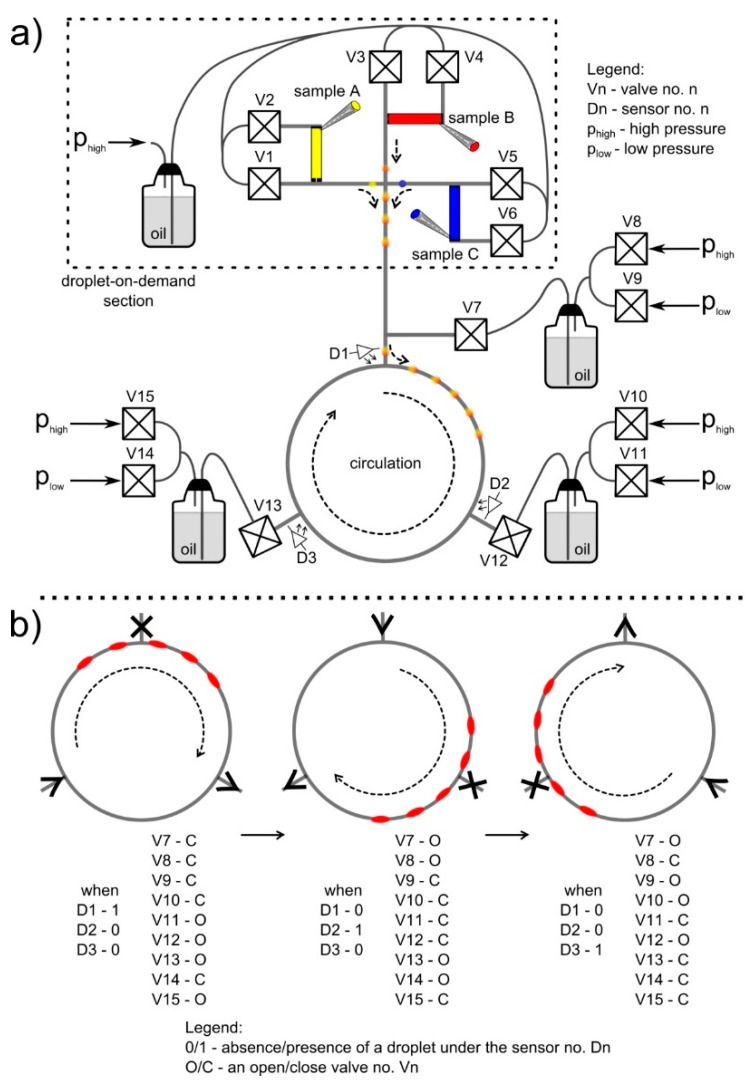
(**a**) Scheme of the microfluidic system. It consisted of the droplet-on-demand section, three parts of the capillary tubing connected to each other (forming loop), and pressurized oil containers with T-junctions. (**b**) The sequential turning of the two-state valves placed on the connectors to pressurized containers provided a constant orientation of the motion of droplets inside the loop. The switching of the valves had been selected in such a way as to ensure the circulation of droplets following the clockwise direction. Arrows on the channels show the direction of oil flow. X indicates no flow.

The droplets during the water phase (ink solutions, bacteria medium, LB broth, or chloramphenicol solutions) were produced and introduced to the loop using the droplet-on-demand (DOD) section [22,37], and built with cubes and tubings (Figure 1g,h and Figure 2a). We applied three droplet-on-demand generators (Figure 2a, droplet-on-demand section) to the system that were equipped with deposition ports for different reagents. Each droplet-on-demand generator had two oil valves. One controlled the oil phase, which cut the droplet at the T-junction and pushed it further, and the second allowed a certain amount of the reagent to be dispensed into the system. In the described experiments, we simultaneously formed three droplets that contained the following: (a) an LB broth, (b) a bacterial culture, and (c) a chloramphenicol to merge them in varying proportions to a droplet with a fixed volume. To obtain high precision in the volume of formed droplets, we used feedback from the camera (uEye UI-3140CP-C-HQ Rev.2, IDS, Mannheim, Germany). It enabled us to stop the flow when formed droplets met the required volume. This approach allowed us to generate a droplet of a given length (volume) based on the signal from the image analysis. As a result, the final droplets were always the same volume, with an accuracy of up to ~0.1%. The so-called unwanted droplets, which were usually at the beginning of the tests, were removed as a result of the disconnection between connector cubes with tubings.

In order to achieve a continuous circulation of droplets, we divided the loop into three identical parts, each a 50 cm long loop of tubing, and connected to each other with pressurized containers of the continuous phase using T-junctions cubes (Figure 2a, Video S1). These three sections were a minimal division of the system that allowed for continuous circulation of droplets, although the number of sections were increased for specific requirements. After the introduction of droplets into the loop, we followed the LabWindows protocol: (a) read the signal from detectors placed before the connector to each part of the loop, (b) switch the output of the system to the end of the section where droplets flow in, (c) switch the source of flow to the end of the section from where droplets flow out, (d) open/close appropriate valves in order to achieve the desired direction of flow of droplets (Figure 2b). The loop enabled continuous circulations of droplets without changing the flow direction of droplets.

At the beginning of each experiment, the whole system was sterilized with an autoclave.

### 2.3. Automation

The central part of the control system was the multifunction electronic device (PCIe-6321, National Instruments (NI), Austin, TX, USA) called a card, which was programmed in LabWindows (National Instruments). Digital outputs of the card were used to control the fifteen bistable valves (V165, equipped with Z070D coils, Sirai, Bussero, MI, Italy), according to convention (high voltage, an open valve; low voltage, a closed valve). Electromagnetic and piezoelectric devices were used as valves. The analogue inputs of the card were used to monitor the signals from the light-to-voltage sensors (TSL257, Taos, Plano, TX, USA) and operated at a frequency 10 kHz. This approach allowed us to use the sensors in the dispersed phase as indicators of droplet flow and detectors of light absorption, but only after the integration of the intensity (power) of light passing through a droplet. The pressure in the oil containers was controlled by employing pressure regulators (Bosch Rexroth PR1-RGP, Lohr, Germany) and monitored with the use of digital manometers (AZ 82100, AZ Instruments, Taichung, Taiwan) connected with PC via RS-232 standard. The temperature was stabilized within the range of 0.1 °C in a thermostatic Styrofoam box. The whole microfluidic system was immersed in distilled water. The flow of the continuous phase was driven by a stable pressure difference (p_high_ − p_low_ = 100 ± 0.01 mbar) between two containers. The generation of droplets was aided with an edge-detection algorithm based on the image analysis [38] provided by the digital camera (uEye UI-3140CP-C-HQ Rev.2, IDS, Mannheim, Germany). The camera tracked the process of droplet generation with a volume accuracy of ~0.1%. The inaccuracy of the detection of droplet edges was 5 μm.

## 3. Results and Discussion

### 3.1. Motion of Fluids in the Loop

The motion of fluids in the microfluidic loop was governed by pressure differences between containers in the continuous phase that were connected with the main loop using T-junctions, and two-state (open/close) bistable valves (Figure 2a). The sequential opening and closing of valves provided a constant orientation of pressure gradients along two segments of the loop. This allowed for an uninterrupted motion of droplets in the system (Figure 2b). The timing of the valves opening and closing was controlled by the electronic feedback loop, which was based on the measurements of droplet position, provided by optical sensors or by the camera. The timing was adjusted dynamically, and the motion of droplets was unaffected by any changes in the rheology of flow due to the altered viscosity (an increase of the hydrodynamicresistance of droplets [39]) during the progress of the reaction. 

As described in the Materials and Methods section, the tubing that connected the main loop with pressure containers was characterized with an ID of 0.8 mm. In comparison to systems that require long thin capillaries to work properly, our solution lowered the hydrodynamic resistance of the whole system [4,22]. Therefore, the pressure difference required to induce motion of the fluids was lower than typical microfluidic devices and equaled ∆p = 100 mbar, which lowered the technical requirements for running the microfluidic loop.

The assembled system was also subjected to tests for pressures higher than 100 mbar. The tightness and leakage of the whole system tested both for air and HFE-7500. To easily visualize the leakage problem, the entire system was immersed in water. As a result, for both air and oil phase, the described connections operated efficiently up to a pressure difference of 2.5 bar. The system had to manage without leakage for at least ten hours to confirm tightness.

### 3.2. Time Evolution of Droplets

**Figure 3:**
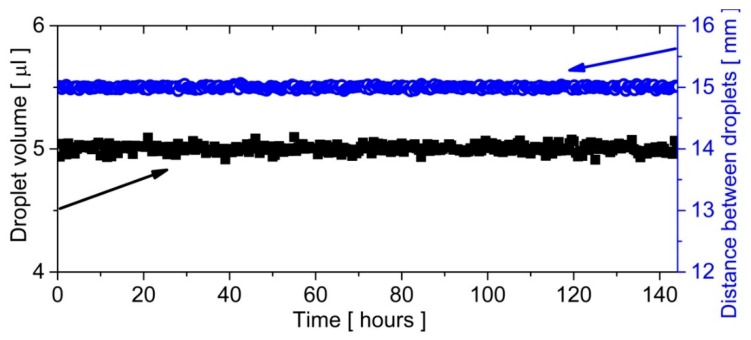
The time evolution of the droplet volume and the distance between droplets circulating in the microfluidic closed loop. For both parameters, the size of the point corresponded to the standard deviation, which was calculated from a sample containing ten droplets flowing in a loop. Since no changes of volume or distance were observed, one can conclude that the motion of droplets in the microfluidic loop was stable even on large time scales.

We measured both the volume and distance between ten droplets flowing sequentially (a train of droplets) in the described loop for 144 h. This translates into 8640 laps of droplets (one lap per minute). The volume of a droplet was estimated from its shape with the use of the image analysis [38]. The droplets never stopped in the loop, and the whole system was immersed in water, where it was thermostated and achieved a stable temperature condition at 37 °C. The obtained results confirmed the constant volumes of droplets as well as a fixed distance between them (Figure 3). The stable mechanical conditions ensured the constant spacing between droplets. Thus, it was not required to use an additional dispersed phase to separate droplets [40]. 

### 3.3. The Bacterial Growth in Closed Loop Circulation

**Figure 4:**
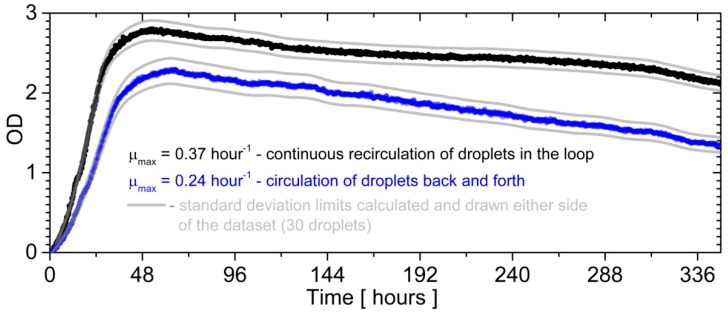
The bacterial growth curve for the long-term incubation of droplets flowing in a microfluidic loop, compared with droplets flowing back and forth in the 1 m long tubing. The optical density (OD) was taken at 600 nm. The black and blue curves represent the average growth calculated from 30 droplets in each case. The maximum growth rate was calculated as a derivative of the OD function in time.

In order to demonstrate the potential of the closed loop circulation for long-term incubation, we cultured bacteria inside droplets during an experimental series. In real-time we measured the optical density (OD) of the sample over the period of 336 h (14 days). The experiment was conducted for thirty droplets (each with a volume of 5 μL) to obtain solid growth curves. To underline the advantages of presented solutions to bacterial culture, we compared the growth of bacteria breeding in 30 droplets that flowed back and forth in 1 m long FEP tubing. The detectors were responsible for the measurement of the optical density and were placed in the middle of tubing, whereas the detectors placed on the edge of the capillary were responsible for measuring the change of oil flow direction. The outcome was that the OD averaged over 30 samples (Figure 4).

As shown in previous reports [39,41,42], the droplets of the dispersed phase moved inside the circular capillary faster than the continuous phase. There was a thin film of oil between a droplet and the wall of the tubing, so that the droplet had a smaller diameter than the diameter of the tubing [42]. Therefore, a droplet’s surrounding is continuously exchanging in circular cross-section channels. This effect was beneficial because the continuous phase was used as a carrier of oxygen or other substances needed to run the reactions inside droplets [43]. It was shown that the continuous phase of oxygenated oil amplified bacterial growth in the water phase [44]. In the presented loop-circulation, droplets had contact with fresh oil, which was continuously changing between containers and tubing. In contrast, the droplets that flowed back and forth in tubing with the same oil was more oxygen-poor after each cycle [43]. Therefore, the droplets that flowed in the loop had a better gas exchange between the water phase and oil phase (Figure 4).

### 3.4. Influence of Chloramphenicol on Bacterial Culture

**Figure 5:**
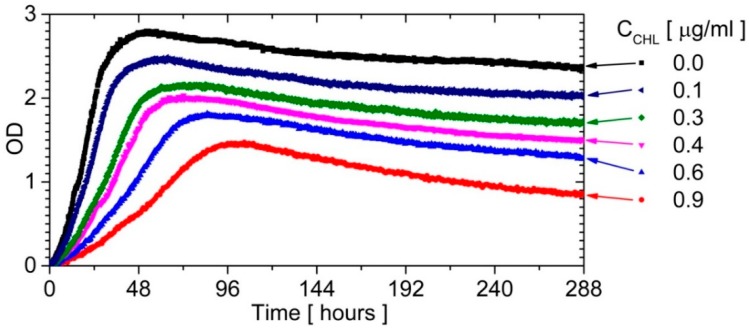
The bacterial growth curves for a long-term incubation inside a droplet for different concentrations of chloramphenicol (CHL).

The experiment was also conducted to discover different antibiotic concentrations in order to determine the effect of its presence on the growth of bacterial culture. We prepared the sequence of 30 droplets, containing chloramphenicol [45] from 0.0 μg/mL to 0.9 μg/mL, with six droplets at each concentration. The recorded growth curves are presented in Figure 5. 

The bacterial strain belonged to a slow-growing strain. So far, no slow-growing strain had been continuously recorded under the following breeding conditions: a gas exchange between the medium in a droplet and oil, as well as excellent mixing in a droplet and stable temperature. The growth curves in Figure 5 show: (a) the rapid adaptation of microorganisms to culture conditions (a short lag phase, almost not observed), (b) the stable exponential phase, (c) the short stationary phase, and (d) the death phase where the rate depends on the antibiotic concentration just like for the exponential phase.

### 3.5. Verifying the Lack of Cross-Contamination of Tubing Walls during the Continuous Phase

We also verified whether the long-lasting presence of microorganisms in droplets lead to the contamination of the inside of capillaries (biofilm formation) or the continuous phase (crossing of the water-oil interface) with biological material. After the removal of the oil in the continuous phase, it was mixed with fresh LB broth and incubated for two days. After incubation, the medium was spread on ten LB agar plates (smear test). In an experiment, no growth of bacteria was observed.

Next, we prepared 30 of 5 μL microdroplets, containing LB growth media, and recirculated them for two weeks with the use of the same oil as in previous experiments. We did not observe any OD increase in any of the 30 droplets that were initially free of bacteria. In addition, when repeating the previous experiments with a different chloramphenicol concentration but the same oil, we reproduced the results in the limit of statistical error.

With the use of the confocal microscopy, the surface of the microchannel was also checked for biological contaminants. During the tests, no remaining biofilm were found in the system.

We conclude that no contamination occurred in the presented system. It was proven that oil in the loop could be reused and adsorption of microorganisms on the tubing wall [46] was absent.

## 4. Conclusions

We have presented the design of a novel circular microfluidic loop, which enables an uninterrupted cycling of a dispersed phase using three pressurized continuous phase containers that support the loop upon the sequential switching of valves. The system is particularly suitable for chemical, biological, and biotechnological experiments (PCR, microorganisms incubation, etc.) as it prevents cross-contamination and the formation of biofilms. This is achieved by using hydrophobic materials in the construction of the loop (Teflon FEP) and enforcing the continuous motion of droplets without any physical contact of a droplet within the channel walls [47]. 

Additionally, the design of our system allows for an increase in the throughput (i.e., the number of droplets circulating in the loop solely by changing the length of the section). The set of three pressurized containers and system of valves that induce the motion of the fluid remain unchanged. This feature provides a simple scaling of the system without building up the laboratory equipment needed for its operation. Therefore, it can be tailored to the specific needs of an experiment. Moreover, the possibility of changing the length of the loop simplifies the construction of setups that require changes of the temperature reaction, like PCR reactions. In such a case, the system can be locally heated by infrared radiation, and the time of heating optimized for the efficiency of the reaction determined solely by the geometry of the system (this, however, is a work in progress).

The system also provides the real-time monitoring of the reaction’s progress. The loop is a facile platform for long-term experiments, including monitoring of antibiotic treatment. We have confirmed in a two-week incubation of bacteria that no contamination occurred to the microfluidic system or the oil acting as the continuous phase. Therefore, there is no need for replacing the oil after the experiment, which reduces the cost of a single experiment down to the cost of water reagents of the reaction and minimizes the risk of spillage of liquids to the environment.

## Supplementary Materials

The following are available online at http://www.mdpi.com/2072-666X/9/9/469/s1, Video S1: The circulation of droplets in a loop.
